# *Notes from the Field:* Rhodesiense Human African Trypanosomiasis (Sleeping Sickness) in a Traveler Returning from Zimbabwe — United States, August 2024

**DOI:** 10.15585/mmwr.mm7409a3

**Published:** 2025-03-20

**Authors:** Elizabeth M. Wendt, Farrell A. Tobolowsky, Gerardo Priotto, Jose Ramon Franco, Rebecca Chancey

**Affiliations:** ^1^Division of Parasitic Diseases and Malaria, National Center for Emerging and Zoonotic Infectious Diseases, CDC; ^2^Georgia Infectious Diseases, PC, Atlanta, GA; ^3^Department of Control of Neglected Tropical Diseases, World Health Organization, Geneva, Switzerland.

**Summary**

**What is already known about this topic?**

Sleeping sickness or human African trypanosomiasis (HAT) is a rare and fatal disease, if left untreated, that is endemic in sub-Saharan Africa.

**What is added by this report?**

A U.S. traveler returning from Zimbabwe in August 2024 developed rhodesiense HAT and was successfully treated after prompt diagnosis. Three additional cases in persons from other countries who traveled to the same region were reported to the World Health Organization. These are the first Zambezi Valley–associated cases reported since 2019.

**What are the implications for public health practice?**

Clinicians should consider rhodesiense HAT in travelers with fever who have recently been in an area where *T.b. rhodesiense* is endemic, even if that area has not reported recent cases of disease. Timely treatment is critical to a favorable outcome.

Human African trypanosomiasis (HAT), also known as sleeping sickness, is a vectorborne disease caused by two subspecies of the parasitic protozoan *Trypanosoma brucei: T.b. gambiense *and *T.b. rhodesiense.* Both are transmitted by the bite of the tsetse fly, but they differ in epidemiology and disease progression. *T.b. gambiense* is found in west and central Africa and accounts for approximately 92% of HAT cases globally, while *T.b. rhodesiense *is found in east and south Africa and is often the cause of HAT in short-term travelers to endemic regions (*1*). *T.b. gambiense *causes a slowly progressive form of HAT, with symptoms developing over months to years, while *T.b. rhodesiense* causes an acute and quickly progressive form of the disease. 

Domestic cattle and several big-game animals are reservoirs for T.b. rhodesiense* (*1*,*2*). During the first stage of rhodesiense HAT, which is rapidly progressive over weeks, the parasite multiplies in the blood and lymphatic system, causing symptoms including fever, hemolysis and anemia, thrombocytopenia, hepatosplenomegaly, and renal disease. In the second stage of disease, the parasite crosses the blood-brain barrier, causing central nervous system (CNS) dysfunction, followed by death within weeks to months (Box). Updated 2024 World Health Organization (WHO) guidelines recommend oral fexinidazole for treatment of first and second stage rhodesiense HAT (*3*). Approximately 90% of cases are fatal without treatment; therefore, prompt identification of trypanosomes and early treatment is critical. Parasite burden is high in rhodesiense HAT; because relapse is possible if treatment fails to eliminate all parasites, WHO recommends follow-up at 3, 6, and 12 months after treatment (*3*).

BOX **Epidemiologic characteristics of human African trypanosomiasis (sleeping sickness) caused by infection with *Trypanosoma brucei* *rhodesiense***

**Parasitic Sub-Species**

*Trypanosoma brucei rhodesiense*
**
**

**Geographic location**

East and southern Africa

**Cases reported in Africa (2023)**

Twenty-four reported in four countries (Ethiopia, Malawi, Tanzania, and Zambia)

**Risk factors**

Travel to wildlife preservations (national parks and game reserves) or game hunting; for local populations, hunting, herding, and cattle raising

**Vector**

Tsetse fly (*Glossina spp.*)

**Primary reservoir**

Domestic cattle, some big-game and safari animals (impala, lion, waterbuck, zebra, giraffe, warthog, and others)

**Incubation period**

Weeks to months

**Prevention**

- Wear thick, neutral-color clothing, including long pants, long-sleeved shirts, and socks because tsetse flies are attracted to bright and contrasting colors
- Inspect vehicles for tsetse flies before entering
- Avoid bushes

**Frequency in persons for countries without endemic disease**

Occasional cases among returning travelers (especially those having visited safari parks or game hunters)

*T.b. rhodesiense* is endemic in 13 countries.^†^ Since 2011, reported rhodesiense HAT cases have been steadily declining, with only 24 cases reported in 2023. Published literature describes fewer than 30 cases in short-term travelers from countries without endemic disease since 2011 (*4**,**5*). Because *T.b. rhodesiense* is maintained in animal rather than human reservoirs, rhodesiense HAT is targeted for elimination as a public health problem rather than for interruption of transmission; however, occasional human cases and localized outbreaks remain possible. This case report describes the clinical course of a traveler who developed symptoms and signs of rhodesiense HAT after returning to the United States from Zimbabwe.

## Case Report and Outcome

In August 2024, CDC was contacted regarding diagnosis and management of a case of HAT caused by *T.b. rhodesiense* in a U.S. traveler aged 57 years who had recently returned from safari in the Zambezi Valley in northern Zimbabwe. The patient was evaluated at a U.S. hospital with a 2-day history of fever and a well-demarcated, ulcerated lesion on the left thigh, approximately 2 weeks after presumed exposure to *T.b. rhodesiense* parasites in an endemic area. He had no neurologic symptoms. A peripheral blood smear, obtained to rule out malaria, revealed parasites consistent with *Trypanosoma brucei* spp., which was confirmed by CDC’s reference laboratory.^§^ The patient’s presenting signs and symptoms and epidemiologic exposure risk were consistent with rhodesiense HAT.

In accordance with WHO guidelines, oral fexinidazole was initiated (*3*). The patient rapidly progressed to multisystem organ failure requiring dialysis and intubation for respiratory distress in the setting of volume overload. Intramuscular pentamidine, an alternative anti-trypanosomal drug that can be used in first stage disease, was added given the uncertainty of fexinidazole absorption by feeding tube. Intravenous suramin, used as first-line treatment for first stage rhodesiense HAT prior to the new guidelines in 2024, is relatively contraindicated in renal impairment.^¶^ The patient remained at neurologic baseline throughout his clinical course, although severe thrombocytopenia, a known complication of rhodesiense HAT, precluded lumbar puncture to confirm absence of CNS involvement (i.e., second stage disease). Ultimately, the patient received 10 days of pentamidine and fexinidazole and was discharged home with only mild renal dysfunction. No signs of relapse were evident 6 months after discharge.

## Preliminary Conclusions and Actions

Between this patient’s presentation in August 2024 and January 2025 three additional cases of rhodesiense HAT were reported to WHO in persons from nonendemic countries who were bitten by a tsetse fly while traveling in the Zambezi Valley. The Zambezi Valley spans northern Zimbabwe and southern Zambia, where epidemiologic conditions are similar, and the parasite is endemic. These four cases are the first Zambezi Valley–associated cases reported since 2019, although Zambia has experienced human cases in other areas during this period.

Clinicians should urgently consider HAT caused by *T.b. rhodesiense *in travelers with fever arriving from an endemic area, even if cases have not been reported from that area recently. Delayed treatment can be fatal, so if rhodesiense HAT is suspected, clinicians should promptly obtain a peripheral blood smear to assess for trypanosomes and consider contacting CDC if diagnostic confirmation or treatment recommendations are needed. 2024 WHO guidelines recommend fexinidazole as first-line treatment for both first and second stage rhodesiense HAT with frequent post-treatment monitoring (*3*). Clinicians requiring assistance with diagnosis or treatment may contact CDC subject matter experts at parasites@cdc.gov or +1-404-718-4745.

## Footnotes

* Although largely asymptomatic in indigenous animals, infection can produce fever, itching, and lymphadenopathy in the first stage, followed by sleep disturbances, poor coordination, and behavior changes in the second stage.

† According to WHO, rhodesiense HAT is endemic in Botswana, Burundi, Eswatini, Ethiopia, Kenya, Malawi, Mozambique, Namibia, Rwanda, Tanzania, Uganda, Zambia, and Zimbabwe.

§ The presence of trypanosomes was confirmed by microscopy.

¶ Intravenous suramin is another therapeutic option but can result in renal toxicity; its use is therefore to be avoided in patients with renal impairment. https://reference.medscape.com/drug/suramin-sodium-suramin-342671#5
